# Sex hormones affect endothelial lipase-mediated lipid metabolism and atherosclerosis

**DOI:** 10.1186/s12944-019-1175-4

**Published:** 2019-12-23

**Authors:** Chuan Wang, Manabu Niimi, Shuji Kitajima, Fumikazu Matsuhisa, Haizhao Yan, Sijun Dong, Jingyan Liang, Jianglin Fan

**Affiliations:** 1 Department of Pharmacology, Shaanxi University of Chinese Medicine, Xianyang, China; 2 Department of Molecular Pathology, Faculty of Medicine, Graduate School of Medical Sciences, University of Yamanashi, 1110 Shimokato, Yamanashi, 409-3898 Japan; 3 Analytical Research Center for Experimental Sciences, Saga University, Saga, Japan; 4 Key Lab of Urban Environment and Health, Institute of Urban Environment, Chinese Academy of Sciences, Xiamen, China; 5 Research Center for Vascular Biology, School of Medicine, Yangzhou University, Yangzhou, China; 6 School of Biotechnology and Health Sciences, Wuyi University, Dongcheng Cun No. 22, Jiangmen, 529020 China

**Keywords:** Atherosclerosis, Hypercholesterolemia, Endothelial lipase, Sex hormones, Gender difference

## Abstract

**Background:**

Endothelial lipase (EL) plays an important role in lipoprotein metabolism and atherosclerosis. To study the functional roles of EL, we recently generated transgenic (Tg) rabbits and reported that increased hepatic expression of EL in male Tg rabbits significantly reduced diet-induced hypercholesterolemia compared with non-Tg controls. This gender difference suggests that sex hormones may mediate EL functions thereby influencing lipoprotein metabolism. To examine this hypothesis, we compared the effects of orchiectomy and ovariectomy on plasma lipids and diet-induced atherosclerosis in both Tg and non-Tg rabbits.

**Methods:**

Male rabbits were under orchiectomy whereas female rabbits were under ovariectomy. We compared plasma lipids, lipoproteins, and apolipoproteins of rabbits before and after surgery in each group fed either a chow diet or cholesterol-rich diet.

**Results:**

On a chow diet, both male and female Tg rabbits showed lower plasma lipids than non-Tg counterparts and this lipid-lowering effect of EL was not affected by either orchiectomy in male or ovariectomy in female Tg rabbits. On a cholesterol diet; however, male Tg rabbits but not female Tg rabbits showed significant resistance to diet-induced hypercholesterolemia and atherosclerosis. The EL-mediated atheroprotective effect was eliminated after orchiectomy in male Tg rabbits. Female Tg rabbits showed similar levels of total cholesterol and lesion size of atherosclerosis compared with non-Tg rabbits and ovariectomy did not affect diet-induced hypercholesterolemia or atherosclerosis.

**Conclusion:**

These results suggest that increased EL protects against diet-induced hypercholesterolemia and atherosclerosis. The beneficial effect of EL was dependent upon the presence of androgenic hormones.

## Introduction

Atherosclerotic complications such as myocardial infarction and stroke are the major cause of morbidity and mortality in the world [1, 2]. Atherosclerosis is related with a number of genetic and environmental factors and its pathogenesis is associated with many risk factors, such as hyperlipidemia, hypertension, diabetes and smoking [3–6]. In addition, many other factors are also involved in the development of atherosclerosis. For example, it is well known that the prevalence of atherosclerosis is lower in women before menopause than men. Epidemiological, clinical, and experimental studies have provided ample evidence that there are gender-specific variations in cardiovascular risks [7–10]. However, after menopause, the risk of atherosclerosis in women begins to rise and even exceed that in men, suggesting that estrogen exerts athero-protective functions through mediation of vascular functions and lipoprotein metabolism, hemostasis and fibrinolysis [11–17]. In spite of this, gender-differences cannot be completely explained by estrogen alone and interactions between sex hormones and other factors also play a role in atherosclerosis although it is not completely understood regarding these factors.

Sex hormones affect may many facets of lipid metabolism through interactions with other regulators. Apolipoprotein E (apoE) and lipoprotein lipase (LPL), two important mediators in lipoprotein metabolism, are significantly affected by sex hormones because male and female animals exhibit different lipoprotein profiles and response to a cholesterol diet and atherosclerosis when these two genes are overexpressed [18, 19]. Endothelial lipase (EL) was originally cloned from endothelial cells [20, 21], but EL is also expressed in liver, lung, thyroid, and kidney [22, 23]. Although EL, LPL, and hepatic lipase (HL) hydrolyze both triglycerides (TG) and phospholipids (PL) in the lipoproteins, they show different substrate selectivity. EL exhibits high phospholipase activity in high-density lipoproteins (HDL) but low triglyceride lipase activity, whereas LPL and HL mainly exercise TG lipase activity [24, 25]. Previous studies have shown that EL mediates HDL-cholesterol (HDL-C) levels along with VLDL levels [21, 26]. Recently, we found that expression of hepatic EL showed a significant influence on plasma lipids and lipoprotein metabolism [27]. Interestingly, increased hepatic expression of EL inhibits cholesterol diet–induced hypercholesterolemia and protects against atherosclerosis in male Tg rabbits but not in female Tg rabbits. This finding prompted us to envision that there is an interaction between EL and sex hormones thereby influencing lipoprotein metabolism and atherosclerosis. To investigate this issue, we compared the lipoprotein features in male and female rabbits after castration. Our current study showed that gender differences in lipid metabolism and atherosclerosis observed in EL Tg rabbits are dependent on the presence of androgens but not estrogens.

## Materials and methods

### Animals

Transgenic (Tg) Japanese white rabbits expressing the human EL in the liver were generated in our laboratory as described previously [27]. Rabbits were fed with a chow diet (RM-4, Funabashi Farmer) containing 16.5% protein, 4.2% fat, and 13% crude fiber. Tg rabbits along with sex- and age-matched non-Tg littermates (4–6 months old) were used for the current study. For a cholesterol diet experiments, all rabbits were fed with a chow diet supplemented with 0.3% cholesterol and 3% soybean oil for 16 weeks. AII animal experiments were performed with the approval of the Animal Care Committee of the University of Yamanashi and Saga and conformed to the Guide for the Care and Use of Laboratory Animals published by the US National Institutes of Health.

### Analysis of plasma lipids and lipoproteins

Plasma levels of total cholesterol (TC), triglycerides (TG) and HDL cholesterol (HDL-C) were measured using enzymatic assay kits (Wako Pure Chemical Industries Ltd., Osaka) [27, 28]. Plasma lipoproteins were further isolated by sequential gradient ultracentrifugation and then subjected to a 1% agarose gel (Helena Laboratories, Saitama, Japan) electrophoresis and stained with Fat Red 7B or transferred to a nitrocellulose membrane for immunoblotting with polyclonal antibodies (Abs) against apoB, apoE (Rockland Inc., Limerick, PA, USA), and apoAI (Bio-Rad AbD Serotec, Kidlington, UK). Lipoprotein fractions were fractionated by 4~20% SDS-PAGE. Apolipoproteins were visualized by Coomassie brilliant blue (CBB) staining. TC and TG contents in each density fraction were measured using the Wako assay kits described above [27, 28].

### Effects of sex hormone on lipids and atherosclerosis

To investigate the effect of sex hormones on EL-mediated lipoprotein metabolism, male Tg rabbits along with non-Tg littermates were undergone a bilateral orchiectomy whereas female Tg rabbits and littermates were undergone a bilateral ovariectomy [19]. All castrated rabbits were further fed a cholesterol-rich diet containing 0.3% cholesterol and 3% soybean oil for 16 weeks and then their plasma lipids and aortic lesions of atherosclerosis were compared. Because only male Tg rabbits showed protection against diet-induced hypercholesterolemia (see below), we envisioned that estrogen may suppress the functions of EL in male rabbits. To investigate this issue, male Tg rabbits and non-Tg littermates at 4 months of age were intramuscularly injected with 17α-ethinyl estradiol (Sigma-Aldrich, St. Louis; USA) at a dose of 100 μg/kg/day for 10 days [19]. After estrogen injection or ovariectomy, blood was collected from fasted rabbits for plasma lipid analysis as described above. Plasma levels of testosterone and estradiol were measured using Electro chemiluminescence immunoassay (ECLIA) (SRL, Inc. Tokyo, Japan). Plasma EL levels were measured using an ELISA kit as described previously [27].

### Quantification of aortic atherosclerosis

At the end of cholesterol diet feeding for 16 weeks, all rabbits were euthanized by injection of an overdose of sodium pentobarbital solution. The whole aortas were stained with Sudan IV for evaluation of the gross lesion size as described previously [27]. For microscopic evaluation of the lesion area, the aortic arch was dissected into 10 sections and embedded in paraffin. Serial sections (3 μm thick) were stained with hematoxylin-eosin (HE), elastica van Gieson (EVG) and immunohistochemically stained with monoclonal Abs against macrophages RAM11 (Dako Inc., Carpinteria, CA) (1:400 dilution) and smooth muscle α-actin HHF35 (Dako Inc., Carpinteria, CA) (1200 dilution). The microscopic lesion area, macrophage and SMC contents in the lesions were quantified with WinRoof image analysis system (Mitani Co., Tokyo, Japan) [27].

### Statistical analysis

All data are expressed as mean ± SD. ANOVA was used for lipid parameter analysis and Mann-Whitney U-test was used for lesion analysis using SPSS 22.0 software. *P* values < 0.05 were considered significant.

## Results

### Plasma lipids and lipoproteins

**Fig. 1:**
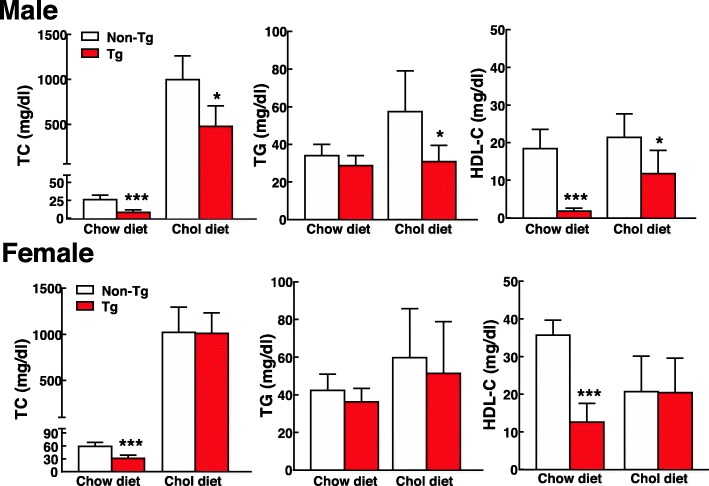
Plasma total cholesterol (TC), triglycerides (TG), and high-density lipoprotein-cholesterol (HDL-C) levels of transgenic (Tg) and non-Tg rabbits fed either a chow diet or a cholesterol-rich diet for 8 weeks. Plasma lipids were analyzed from fasting EDTA plasma of both male and female rabbits. *n* = 5 for male non-Tg and Tg rabbits; *n* = 11 for female non-Tg and *n* = 13 for female Tg rabbits. Data are expressed as mean ± SD. *p < 0.05 and ***p < 0.001 vs non-Tg rabbits

On a chow diet, both male and female Tg rabbits showed significant reduction of plasma total cholesterol mainly caused by significant decrease of HDL-C levels (Fig. 1). Plasma levels of TC were reduced by 68% in males and 47% in females while HDL-C levels were reduced by 90% in males and 65% in females compared with non-Tg counterparts. Plasma TG levels were not significantly changed in Tg males and females compared with non-Tg rabbits.

On a cholesterol diet for 16 weeks; however, male Tg rabbits exhibited protection against diet-induced hypercholesterolemia: TC, TG and HDL-C levels of Tg rabbits remained lower than those of non-Tg rabbits (Fig. 1). In contrast, in female Tg rabbits, TC, TG and HDL-C levels were not significantly different from those of non-Tg groups after a cholesterol diet feeding (Fig. 1).

In the previous study, we showed that there was a marked change in male Tg rabbits compared with non-Tg rabbits: there was a remarkable reduction of apoB-containing particles associated with reduced contents of apoB and apoE as shown in the previous studies [27].

**Fig. 2:**
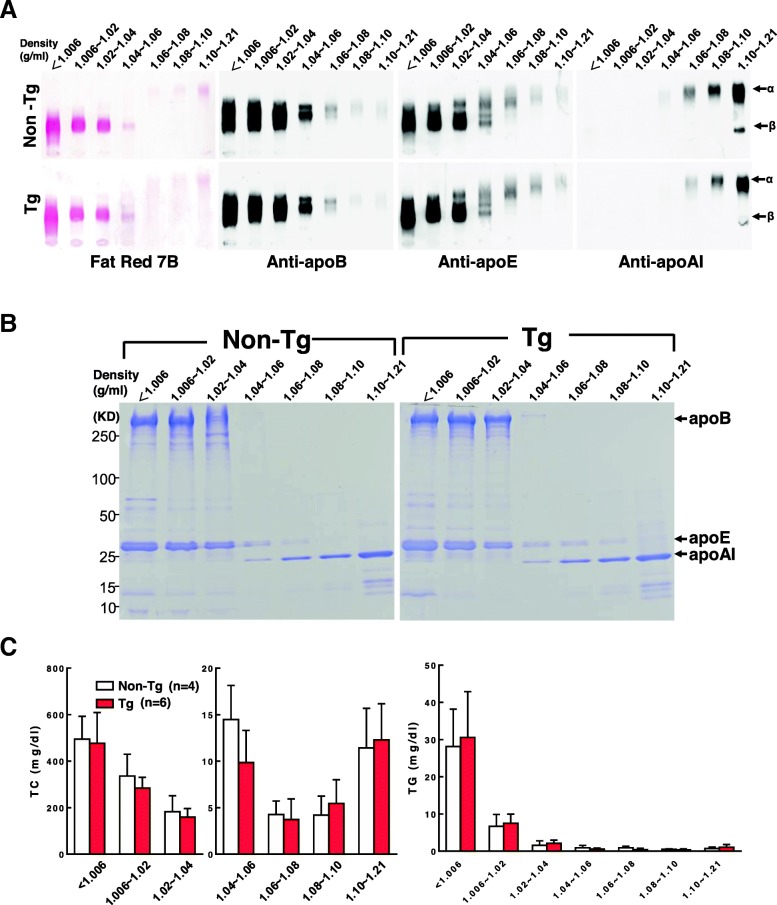
Analysis of plasma lipoproteins isolated from female rabbits at 15 week after cholesterol diet feeding. An equal volume (2 μL) of each fraction was resolved by electrophoresis in a 1% agarose gel. Lipoproteins were visualized using Fat Red 7B staining, and apolipoproteins were identified by immunoblotting with apoB, apoE, and apoAI Abs (**a**). An equal volume of each fraction (5 μL) was resolved by electrophoresis by 4 to 20% SDS-PAGE. Apolipoproteins were visualized using Coomassie brilliant blue staining (**b**). The quantitation of cholesterol and TG contents in lipoprotein fractions of rabbits fed a cholesterol-rich diet for15 weeks (**c**). TC and TG contents in each density fraction were quantified. Data are expressed as mean ± SD

However, female Tg rabbits showed a similar lipoprotein profile including apoB and apoE contents in apoB-containing particles and apoAI and apoE in HDL particles compared with non-Tg females (Fig. 2a–b). Quantitation of TC and TG contents in these fractions showed that all lipoproteins were similar in female Tg rabbits compared with non-Tg females (Fig. 2c).

These observations suggest that sex hormones may affect the functions of EL in Tg rabbits on a cholesterol diet.

### Effects of castration on plasma lipids and atherosclerosis

**Fig. 3:**
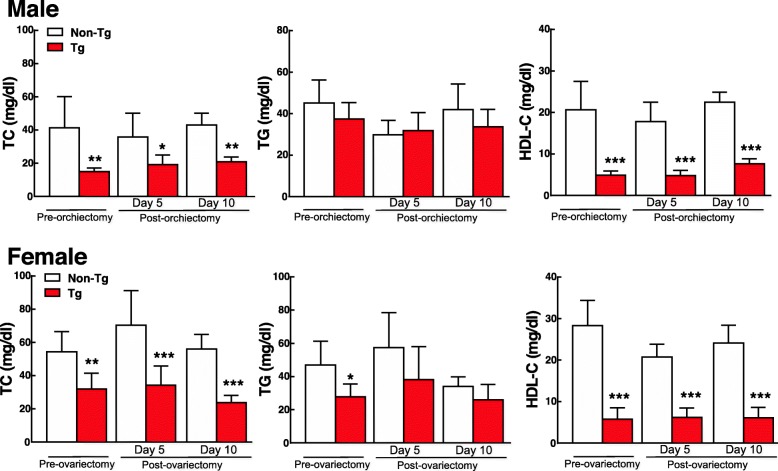
Plasma TC, TG and HDL-C levels of Tg and non-Tg rabbits before and 5 or 10 days after orchiectomy (male) or ovariectomy (female). *n* = 5 for male group, and *n* = 8 non-Tg female and *n* = 4 for Tg female rabbits. Data are expressed as mean ± SD. *p < 0.05, **p < 0.01, ***p < 0.001 vs non-Tg rabbits

**Fig. 4:**
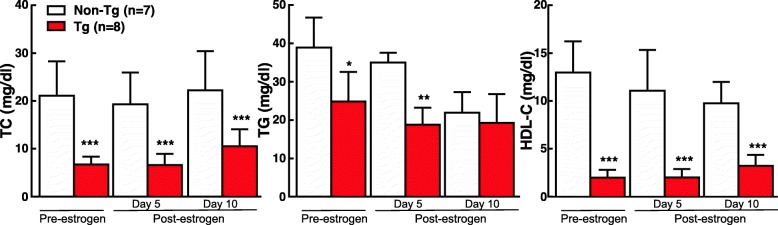
Plasma lipids TC, TG and HDL-C of male Tg and non-Tg rabbits that were injected intramuscularly with 17β-ethinyl estradiol at a dose of 100 μg/ kg/day for 10 days. *n* = 7 for male non-Tg rabbits and *n* = 8 for male Tg rabbits.. Data are expressed as mean ± SD. *p < 0.05, **p < 0.01, ***p < 0.001 vs non-Tg rabbits

To determine if ablation of endogenous androgen or estrogen would affect EL-mediated lipoprotein metabolism and atherosclerosis, we examined the changes of male rabbits that were undergone a bilateral orchiectomy and female rabbits that were undergone a bilateral ovariectomy. Orchiectomy dropped the plasma levels of testosterone (1.15 ng ± 0.44 ng/ml to 0.25 ± 0.06 nm/ml after orchiectomy, *n* = 6 for each group) and elevated plasma EL in male Tg rabbits on a chow diet (Additional file 1: Figure S1). Ovariectomy did not change the plasma levels of EL in female Tg rabbits on a chow diet but elevated EL when they were on a cholesterol-rich diet. Regardless of this, as shown in Fig. 3, castration did not affect the plasma lipids in both male and female rabbits on a chow diet. Plasma levels of TC and HDL-C remained at lower in Tg rabbits compared with non-Tg counterparts. We were particularly interested in male Tg rabbits because they showed protection against diet-induced hypercholesterolemia (Fig. 1). We further intramuscularly injected male rabbits with 17α-ethinyl estradiol for 10 days. Similar to orchiectomied male rabbits, estrogen injection in male rabbits did not change the patterns of plasma lipids compared with un-injected rabbits (Fig. 4). These results suggest that sex hormones did not directly influence EL-mediated lipid metabolism in Tg rabbits on a chow diet.

**Fig. 5:**
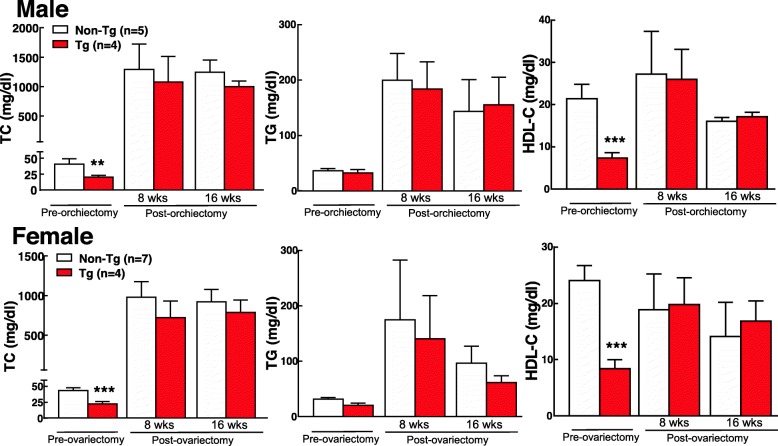
Plasma total cholesterol (TC), triglycerides (TG), and high-density lipoprotein-cholesterol (HDL-C) levels of transgenic (Tg) and non-Tg rabbits fed a cholesterol-rich diet for 16 weeks after orchiectomy (male) or ovariectomy (female). Data are expressed as mean ± SD. **p < 0.01, ***p < 0.001 vs non-Tg rabbits

Then, we fed castrated rabbits with a cholesterol-rich diet for 16 weeks and compared their plasma lipids with uncastrated controls. Interestingly, castrated Tg male rabbits showed similar “high” hypercholesterolemia compared with non-Tg rabbits: TC, TG and HDL-C levels were basically similar to those of non-Tg rabbits (Fig. 5). In another word, EL-mediated protection against hypercholesterolemia in male Tg rabbits was totally eliminated by castration. On the contrary, castrated female Tg rabbits had similar levels of lipids compared to non-Tg rabbits, as those uncastrated female rabbits: there was no significant difference in plasma lipids of Tg rabbits and non-Tg rabbits regardless of castration (Fig. 5).

**Fig. 6:**
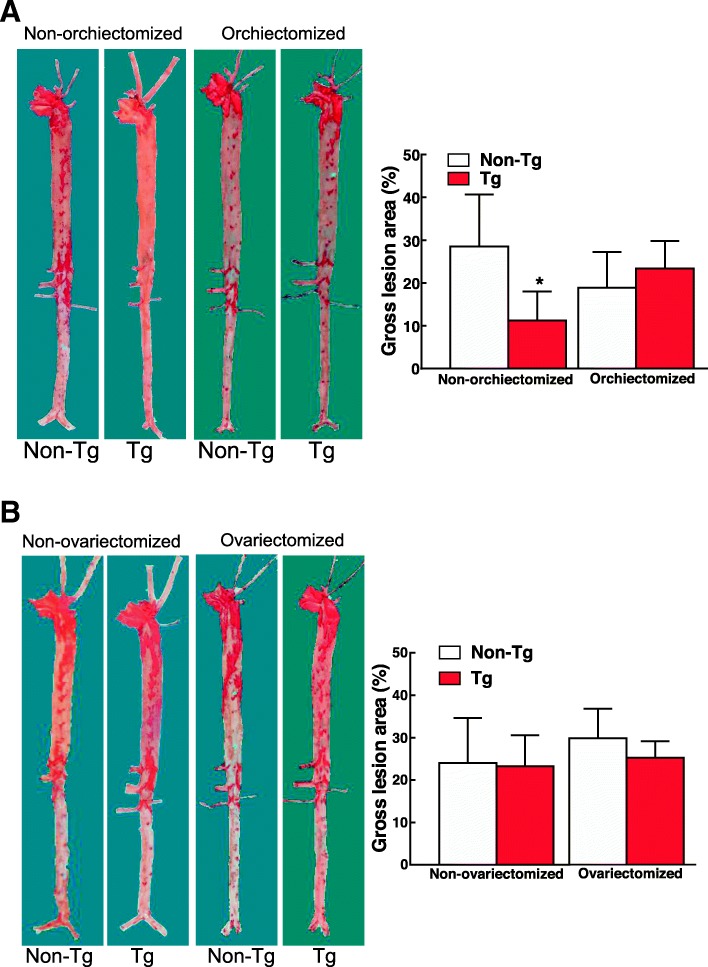
Analysis of atherosclerotic lesions of aorta. Both uncastrated and castrated of Tg and non-Tg rabbits were fed a cholesterol diet for 16 weeks and then the aortic lesions were quantified (**a**  for males and **b** for females). Representative pictures of aortas stained with Sudan IV are shown on the left. The lesion area (defined by the sudanophilic area) was quantified using an image analysis system on the right. *p < 0.05 vs. non-Tg rabbits

Finally, we analyzed *en face* aortic lesion areas and found that the whole aortic atherosclerotic lesions of male Tg rabbits were significantly reduced by 61% in the total aorta compared with non-Tg rabbits. However, after castration, male Tg rabbits showed similar lesion size to that of non-Tg rabbits. This result suggests that castration eliminated atheroprotective effects of EL in male Tg rabbits because there was no significant difference in both plasma TC and aortic atherosclerosis between castrated Tg rabbits and non-Tg rabbits (Fig. 6a). In female rabbits; however, Tg showed similar atherosclerosis lesion area compared with non-Tg rabbits both in uncastrated and castrated rabbits (Fig. 6b). Microscopically, the lesions of atherosclerosis were not significantly changed including the lesion size and cellular components after castration (data not shown).

## Discussion

In the current study, we attempted to elucidate whether sex hormones affect EL-mediated lipoprotein metabolism and atherosclerosis in rabbits fed both chow and cholesterol diets. This study was performed based on the previous observation that increased EL expression in male Tg rabbits protected against cholesterol-induced hypercholesterolemia and atherosclerosis [27]. We found that lipoprotein changes were more prominent in male Tg than female Tg rabbits even on a chow diet. In addition, male but not female Tg rabbits showed protection against cholesterol diet induced hypercholesterolemia and aortic atherosclerosis. This gender difference in terms of EL functions was initially surprising but also reported in EL KO mice. Ishida et al. reported that LDL-C levels were increased by 90% in male LIPG^−/−^ mice as compared with wild-type mice, but no change in females [26]. They showed a significant 24% decrease in the lesion area of male LIPG^−/−^*/*apoE^−/−^ mice when compared with apoE^−/−^ mice; however, the difference was smaller and not statistically significant between female LIPG^−/−^*/*apoE^−/−^ and apoE^−/−^ mice [29].

As we reported in the previous studies, male Tg rabbits showed constantly and significantly lower levels of TC and HDL-C on both chow and cholesterol-rich diets [27]. In HDLs, phospholipids contents were remarkably reduced in both male and female Tg rabbits [27]. In contrast, female Tg rabbits showed lower levels of plasma TC and HDL-C but only on a chow diet. When female Tg rabbits were challenged with a cholesterol diet, they developed similar “high” hypercholesterolemia as non-Tg rabbits. Therefore, EL-mediated cholesterol-lowering and atheroprotective effects were only present in male Tg rabbits, raising a possibility that sex hormones may be involved in EL-lipoprotein metabolism especially on a cholesterol diet.

We initially hypothesized that either estrogen inhibits EL functions or androgen enhances EL functions. To answer these two questions, we performed orchiectomy in males and ovariectomy in females. Our current studies showed that it is mostly likely that the presence of androgen in male Tg rabbits is required for the lipid-lowering and atheroprotective effects of EL because orchiectomy can completely eliminate these beneficial effects of EL. On the other hand, estrogen, if any, has very little influence on EL functions because ovariectomy in females or estrogen treatment in males did not change the lipoprotein profiles and diet-induced hypercholesterolemia and atherosclerosis. In spite of this observation, it is currently unknown how androgen interacts with EL and further studies are needed to elucidate molecular mechanisms. In our previous study, we did not find any difference of EL concentrations of post-heparin plasma between male and female Tg rabbits, suggesting that androgen or estrogen does not directly mediate EL expression [27]. We have found that lipid-lowering functions of EL are essential for atheroprotective effects in male Tg rabbits (Niimi et al. submitted for publication). In light of these findings, there is another possibility that androgen may help promote the hepatic catabolism of apoB-containing particles in male Tg rabbits because we recently showed that Dil-labeled β-VLDL clearance was faster in Tg rabbits than non-Tg rabbits. How androgen interacts with EL; however, requires future investigations. There is a need to delineate the underlying molecular mechanisms of androgen-EL-lipoprotein metabolism. It is well known that sex hormones mediate lipid metabolism and cholesterol biology through a number of pathways [4, 18, 30–32]. Although it is generally believed that androgen such as testosterone may increase men’s cardiovascular risk, it is not clear whether androgen has some other beneficial effects on lipoprotein metabolism in humans. In support of speculation, it has been reported that men with lower testosterone levels tend to have higher incidence of coronary artery disease [33, 34].

## Conclusion

In conclusion, our study has shown that increased expression of EL inhibits diet-induced hypercholesterolemia and atherosclerosis in a gender-dependent manner. Although the molecular mechanisms remain to be defined, it seems that androgen is involved in the lipid-lowering and atheroprotective effects of EL in Tg rabbits.

## Supplementary information

**Additional file 1: Figure S1.** Comparison of EL concentrations after castration in male and female rabbits. Rabbits were either on a chow diet or cholesterol-rich diet. Post-heparin plasma was collected and EL concentrations in pre- and post-orchiectomy or ovariectomy were measured using an EL ELISA kit from IBL (Gunma, Japan). Data are expressed as mean ± SD. *n* = 3–4 for each group.

## Data Availability

The datasets used and/or analysed during the current study are available from the corresponding author upon request.

## Abbreviations

apoE: Apolipoprotein E
EL: Endothelial lipase
HDL-C: HDL-cholesterol
LDL-C: Low-density lipoprotein–cholesterol
LPL: Lipoprotein lipase
PL: Phospholipids
TC: Total cholesterol
Tg: Transgenic
TG: Triglycerides
VLDL: Very low-density lipoprotein
