# Supplementary material for: Sex hormones affect endothelial lipase-mediated lipid metabolism and atherosclerosis
Source: Lipids Health Dis. 2019 Dec 23;18:226. doi: 10.1186/s12944-019-1175-4 (PMC6929444; doi:10.1186/s12944-019-1175-4)
Supplement: Supplementary file 1 — Additional file 1: Figure S1. Comparison of EL concentrations after castration in male and female rabbits. Rabbits were either on a chow diet or cholesterol-rich diet. Post-heparin plasma was collected and EL concentrations in pre- and post-orchiectomy or ovariectomy were measured using an EL ELISA kit from IBL (Gunma, Japan). Data are expressed as mean ± SD. n = 3–4 for each group. [file 12944_2019_1175_MOESM1_ESM.docx]

**EL (pg/ml)**

4000

3000

2000

1000

0

**Male**

**Non-Tg**

**Tg**

10 days

Chow diet

**Post-orchiectomy**

**Pre-orchiectomy**

11 wks

Chol diet

0 day

Chow diet

**Female**

**Non-Tg**

**Tg**

**EL (pg/ml)**

4000

3000

2000

1000

0

5000

Figure S1. Comparison of EL concentrations after castration in male and female rabbits. Rabbits were either on a chow diet or cholesterol-rich diet. Post-heparin plasma was collected and EL concentrations in pre- and post-orchiectomy or ovariectomy were measured using an EL ELISA kit from IBL (Gunma, Japan). Data are expressed as mean±SD. n=3-4 for each group.

10 days

Chow diet

**Post-orchiectomy**

**Pre-orchiectomy**

11 wks

Chol diet

0 day

Chow diet
